# Editorial

**DOI:** 10.1098/rsif.2025.0341

**Published:** 2025-06-11

**Authors:** Richard Cogdell

**Affiliations:** ^1^ Editor-in-Chief

As many of you who interact frequently with *J. R. Soc. Interface* may know, our Founding Managing Editor, Dr Tim Holt, has retired. Tim has guided our journal through its first 20 years. His care and attention to the needs of both authors and reviewers have been one of the reasons why *J. R. Soc. Interface* is held in such high esteem. I want to thank Tim for all the hard work he has done for *J. R. Soc. Interface*, and personally, for his friendship. What many of you may not know is that Tim is a world expert on beer. He has travelled widely to visit various breweries and beer festivals, and he has also written about them. One of the real special pleasures that Tim organized after our Editorial Board meetings was a trip to a local real ale pub where he hosted an evening of beer tasting. Each beer was introduced, and its origins were fully explained. So, Tim, we will miss you for lots of reasons and want to wish you a long, healthy and happy retirement. You will be a hard act to follow.

